# Promising PEGylated cationic dendrimers for delivery of miRNAs as a possible therapy against HIV-1 infection

**DOI:** 10.1186/s12951-021-00899-0

**Published:** 2021-05-28

**Authors:** E. Royo-Rubio, I. Rodríguez-Izquierdo, M. Moreno-Domene, T. Lozano-Cruz, F. J. de la Mata, R. Gómez, M. A. Muñoz-Fernández, J. L. Jiménez

**Affiliations:** 1 Laboratorio InmunoBiología Molecular, Hospital General Universitario Gregorio Marañón (HGUGM), Instituto Investigación Sanitaria Gregorio Marañón (IiSGM), Spanish HIV HGM BioBanco, Madrid, Spain; 2 Plataforma de Laboratorio (Inmunología), HGUGM, IiSGM, Spanish HIV HGM BioBank, Madrid, Spain; 3 Laboratorio Dosimetría Biológica, HGUGM, IiSGM, Madrid, Spain; 4 Departmento Química Orgánica Y Química Inorgánica E Instituto de Investigación Química “Andrés M. del Río″ (IQAR), Universidad de Alcalá (IRYCIS), Campus Universitario, 28871 Madrid, Spain; 5 Networking Research Center On Bioengineering, Biomaterials and Nanomedicine (CIBER-BBN, Madrid, Spain

**Keywords:** Carbosilane dendrimers, microRNAs, HIV-1 infection, Delivery, Inhibition

## Abstract

**Background:**

The appearance of resistance against new treatments and the fact that HIV-1 can infect various cell types and develop reservoirs and sanctuaries makes it necessary to develop new therapeutic approaches to overcome those failures.

**Results:**

Studies of cytotoxicity, genotoxicity, complexes formation, stability, resistance, release and particle size distribution confirmed that G2-SN15-PEG, G3-SN31-PEG, G2-SN15-PEG-FITC and G3-SN31-PEG-FITC dendrimers can form complexes with miRNAs being biocompatible, stable and conferring protection to these nucleic acids. Confocal microscopy and flow cytometry showed effective delivery of these four dendrimers into the target cells, confirming their applicability as delivery systems. Dendriplexes formed with the dendrimers and miRNAs significantly inhibited HIV-1 infection in PBMCs.

**Conclusions:**

These dendrimers are efficient delivery systems for miRNAs and they specifically and significantly improved the anti-R5-HIV-1 activity of these RNA molecules.

**Graphic Abstract:**

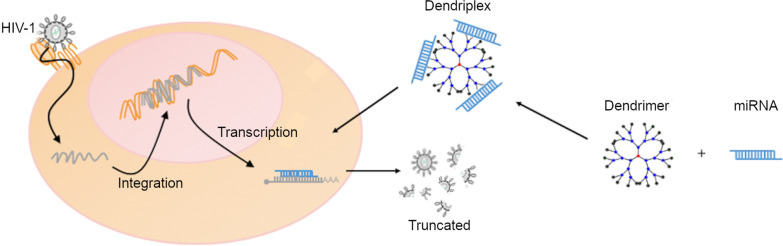

## Background

Human Immunodeficiency Virus (HIV) infection mainly affects CD4 + T lymphocytes, dendritic cells and macrophages [1]. Moreover, there is a wide range of cellular types susceptible to HIV-1 infection, such as central nervous system (CNS) cells including microglia or astrocytes [2]. Lymphocytes are mainly located in the circulatory system and lymphatic ganglions whereas dendritic cells and macrophages play a key role in the immune surveillance of mucosa and tissues. Current treatments against HIV-1 infection control viral replication and disease progression. These treatment combinations mainly include integrase inhibitors such as abacavir, lamivudine, tenofovir alafenamide or emtricitabine, or nucleoside reverse transcriptase inhibitors (NRTIs) such as bictegravir, dolutegravir or raltegravir [3, 4]. Combined therapy includes at least three of these drugs reducing the appearance of mutations and increasing the virological failure. However, the appearance of resistance against these new treatments, mainly in low- and middle-income countries [5–7] and the fact that HIV-1 can infect a wide range of cells and develop sanctuaries and reservoirs [8–11] bring to light the need of new possible therapeutic approaches to eliminate those failures.

Different delivery nanocarriers provide a scaffold through which different small RNA molecules have been successfully delivered in diverse studies [12–15]. We have carried out broad research about various dendrimers in the context of HIV-1 infection [16–20]. Previously, we reported amino-terminated carbosilane dendrimers that provide stability, protection and high transfection efficiency to oligodeoxynucleotides and siRNAs inhibiting HIV-1 replication in peripheral blood mononuclear cells (PBMCs) and CD4 T-cells [21–24]. Carbosilane dendrimers are three-dimensional hyperbranched molecules capable of forming complexes by electrostatic interactions between the positively charged functional terminal groups and negatively charged backbone of small RNAs, what confers protection and facilitates transfection of bound RNAs [25].

Novel therapeutic strategies are being developed from the study of HIV-host interactions, for example microRNAs able to target HIV-1 infection and replication [26]. These miRNAs are small non-coding RNAs, 19–25 long nucleotides, with capability to modulate gene expression at post-transcriptional level [27]. A wide range of different cellular miRNAs has been described to have a negative impact on HIV-1 replication through repressing its expression by targeting HIV-1 3’-UTR or enhancing HIV-1 mRNA interactions with RISC complex [28–30]. The effective delivery of these miRNAs is a limiting factor in their therapeutic applications since naked miRNAs face several difficulties when navigating the circulatory system such as phagocytosis, enzymatic degradation or protein aggregation [31]. Nanotechnology is a promising strategy for the delivery of small RNAs. In this work, G2-SN15-PEG, G3-SN31-PEG, G2-SN15-PEG FITC and G3-SN31-PEG FITC with PEG modifications, were selected. These four carbosilane dendrimers present different PEGylation residues to reduce toxicity and improve the biocompatibility of molecules [32]. Our objective was to form cationic dendrimers-miRNAs complexes that improved the therapeutic effect of current treatments against HIV-1 infection in different cells. We report the potential therapeutic effect of complexation of PEGylated G2-SN15-PEG, G3-SN31-PEG, G2-SN15-PEG-FITC and G3-SN31-PEG-FITC cationic dendrimers with hsa-miR-29a-3p, hsa-miR-29b-3p, hsa-miR-92a-3p, hsa-miR-133b and hsa-miR-149-5p miRNAs in peripheral blood mononuclear cells (PBMCs) and U87MG-CD4^+^CCR5^+^ cell line with anti-HIV-1 activity [33, 34]. First, the biosafety of four cationic dendrimers in PBMCs and l cell line were assessed by measuring cytotoxicity and genetic safety. We characterized several parameters of dendrimers-miRNAs (dendriplexes) formed to determine their stability, resistance to RNases and release capability. The internalization effectiveness of FITC-labelled dendrimers in PBMCs and U87MG-CD4^+^CCR5^+^ cell line was studied. Finally, HIV-1 inhibition capacity of dendriplexes in PBMCs and U87MG-CD4^+^CCR5^+^ cell was determined.

## Results

### Cytotoxicity of the dendrimers on PBMCs and U87MG-CD4^+^CCR5^+^ cell line

**Fig. 1:**
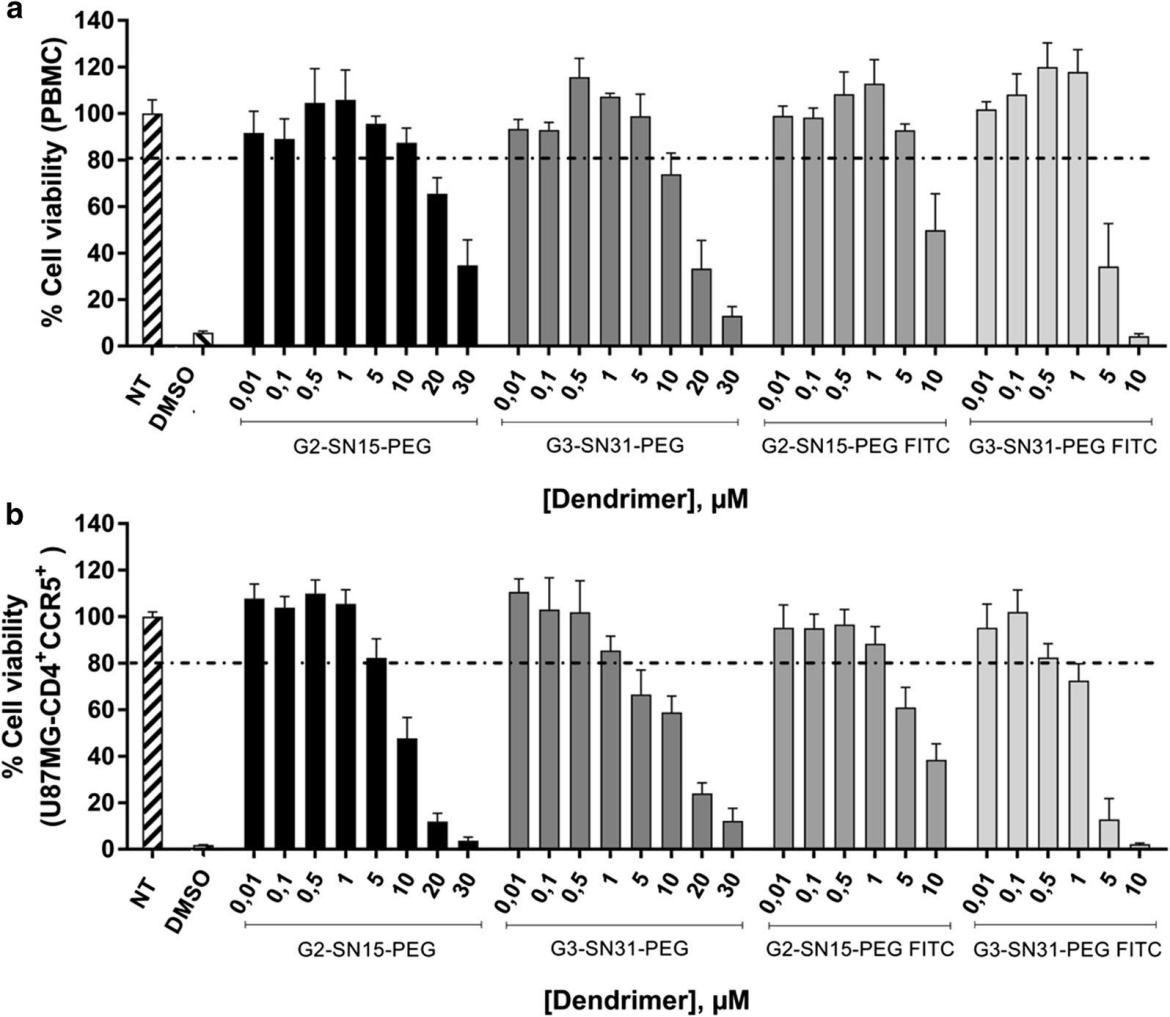
Cytotoxicity of cationic carbosilane dendrimers by MTT assay. PBMCs (**a**) and U87MG-CD4^+^CCR5^+^ cells (**b**) were treated in a range of concentrations from 0.01 to 30 µM of each cationic dendrimer. Cell viability > 80% was established as non-toxic concentration. Culture medium samples were used as cell viability control and DMSO 10% was used as death control. Data represented as mean ± SD of three individual experiments performed in triplicate. *DMSO* dimethyl sulfoxide 10%, *NT* non-treated

Cytotoxicity of G2-SN15-PEG, G3-SN31-PEG, G2-SN15-PEG FITC and G3-SN31-PEG FITC cationic dendrimers in PBMCs and U87MG-CD4^+^CCR5^+^ cells was evaluated analyzing the mitochondrial toxicity by the 3-(4–5-dimethylthiazol-2-yl)-2,5-diphenyltetrazolium bromide (MTT) assay. PBMCs and U87MG-CD4^+^CCR5^+^ cells were treated with increasing concentrations of dendrimers, ranging from 0.01 to 30 µM, for 72 h. Culture medium was used as non-treated control and DMSO 10% as death control. Non-toxic concentrations were considered when the survival rate was ≥ 80%. Our data indicated that G2-SN15-PEG, G3-SN31-PEG, G2-SN15-PEG FITC and G3-SN31-PEG FITC dendrimers were non-toxic in PBMCs at concentrations of 10, 5, 5 and 1 µM, respectively (Fig. 1a) and non-toxic in U87MG-CD4^+^CCR5^+^ cells up at concentrations of 5, 1, 1 and 0.5 µM, respectively (Fig. 1b).

### Genotoxicity of cationic dendrimers

**Fig. 2:**
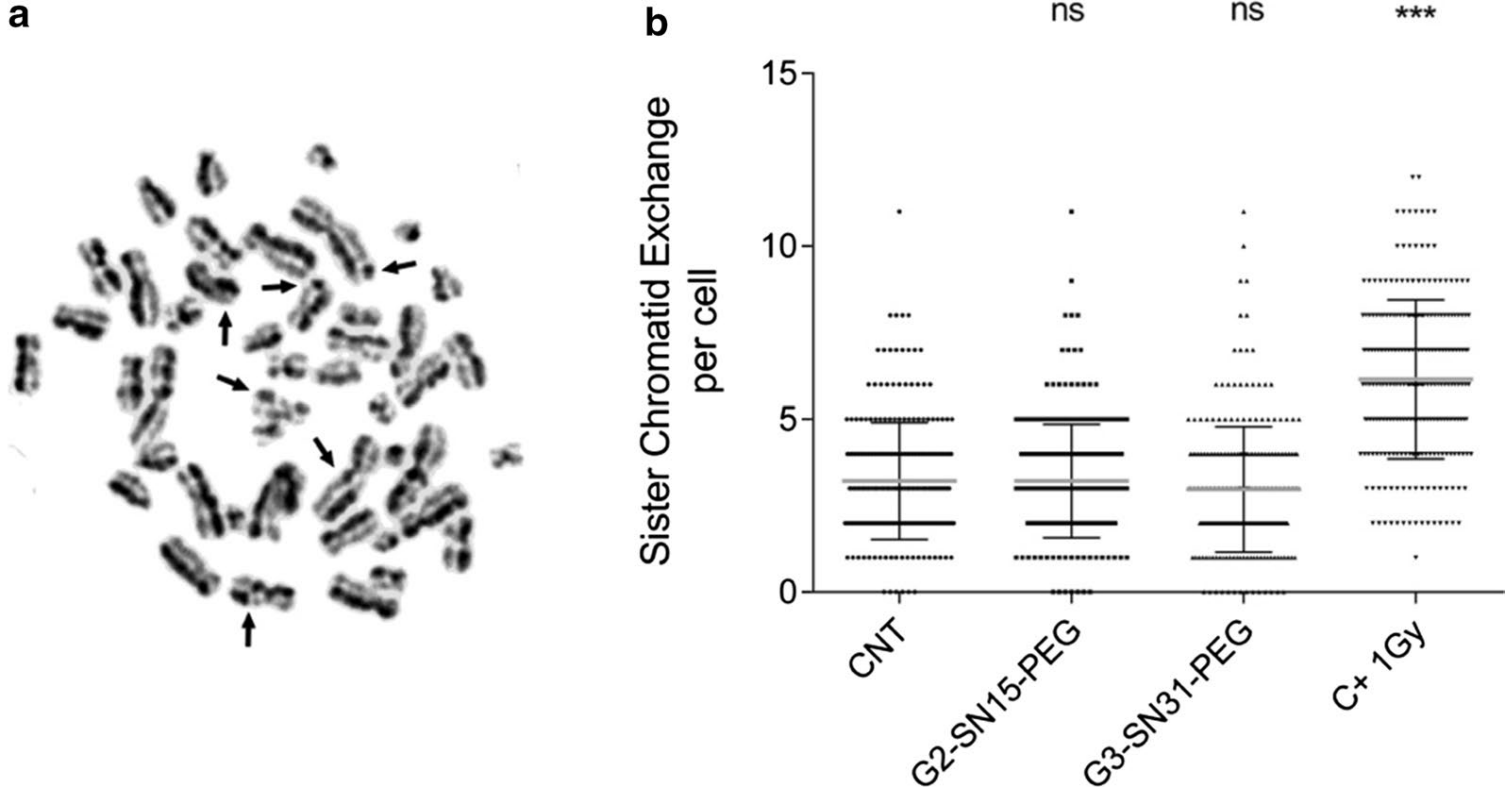
Genotoxicity of carbosilane cationic dendrimers. Quantification of the number of sister-chromatid exchange per cell in PBMCs non-treated, PBMCs treated with G2-SN15-PEG 10 µM, G3-SN31-PEG 5 µM or 1 Gy of radiation for 72 h. **a** Images of random field obtained from the SCE assay after FPG staining. Arrows indicated the sister-chromatid exchange. **b** Analysis of the SCE assay. Data are represented as dot plots of five individual experiments (50 mitosis analysis per healthy donor). *C + 1 Gy* 1 Gy radiation control, *NT* non-treated. (*p < 0.05; **p < 0.01; ***p < 0.001)

If dendrimers generated genotoxicity by interfering with the host genome by sister-chromatid exchange (SCE) assay was studied. It is important to note that dendrimers can bind to miRNAs as well as to host genome due to their positively charged functional groups. If these cationic dendrimers mistakenly targeted the host genome it could cause dangerous genetic changes to its stability. Therefore, it is recommended to perform genetic studies to rule out this event. The SCE assay detects the physical exchange of DNA that occurs between two identical chromatids in the chromosome, determining the genetic damage caused by treatments which persist after the DNA duplication [35–37]. Due to the fact that only fluorochrome unlabeled cationic dendrimers can be used in some studies, genotoxicity tests for G2-SN15-PEG and G3-SN31-PEG cationic dendrimers were performed. PBMCs from five healthy donors were treated with G2-SN15-PEG 10 µM or G3-SN31-PEG 5 µM carbosilane cationic dendrimers for 72 h in presence of 5-bromo-2'-deoxyuridine (BdrU) following fluorescent plus Giemsa stain (FPG) (Fig. 2a). Non-treated samples and a dose of 1 Gy of radiation were used as untreated and genotoxicity control, respectively. Carbosilane cationic dendrimers did not generate genetic toxicity in PBMCs, since there are no significant differences among the non-treated and treated control samples, contrarily to what happens in genotoxicity control studied (Fig. 2b; Table 1).

**Table 1:** Analysis of the genotoxicity of the dendrimers

Treatment	SCE (mean ± SD)	P value	n
CNT	3.22 ± 1.69	-	250
G2-SN-15-PEG	3.22 ± 1.65	1.000	250
G3-SN31-PEG	2.98 ± 1.82	0.1272	250
C + 1 Gy	6.15 ± 2.29	< 0.0001	250

### Dendriplexes formation and characterization

**Fig. 3:**
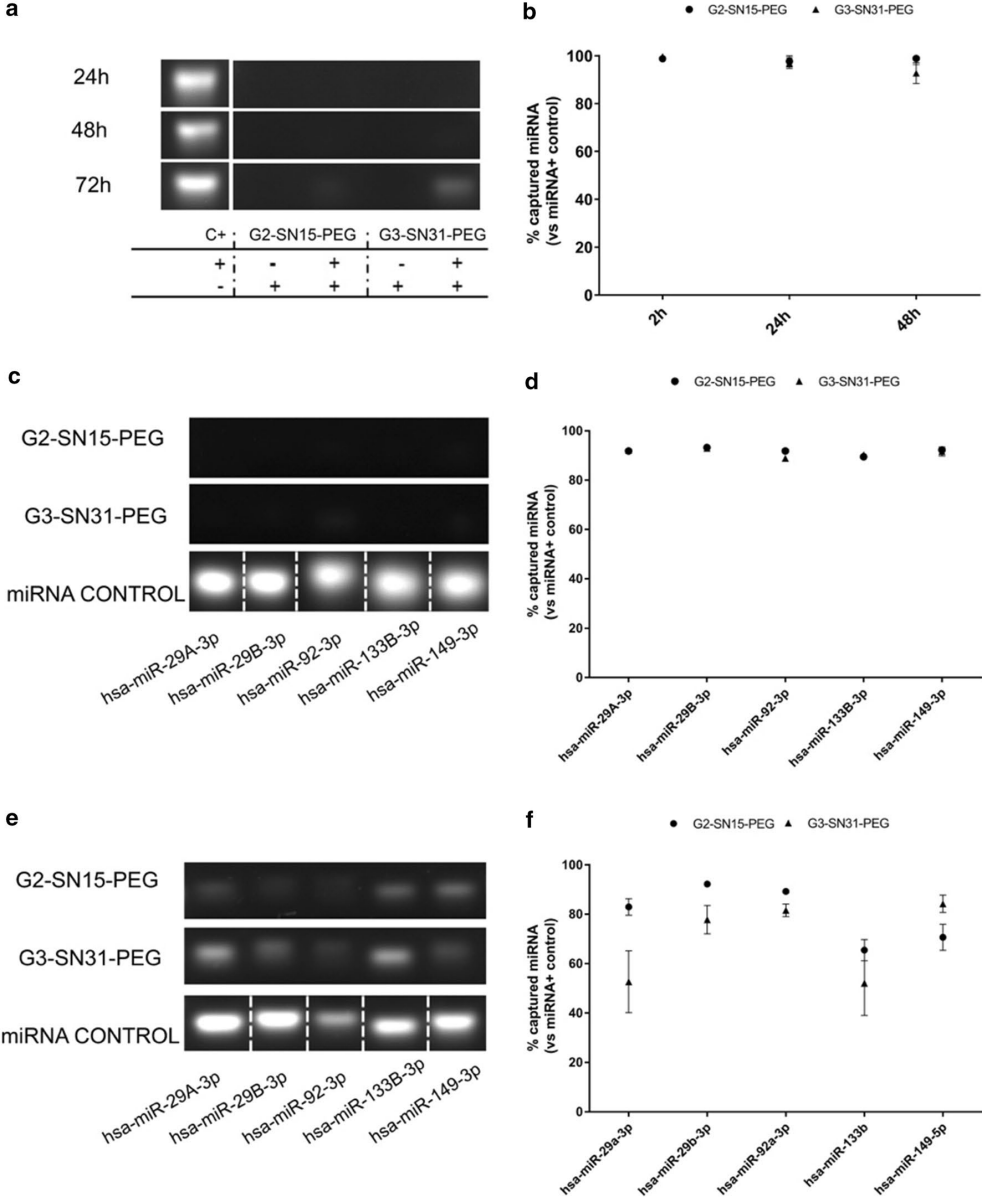
Dendriplexes characterization. **a** Two per cent agarose gel electrophoresis showing the formation of different dendriplexes after 2 h, 24 h or 48 h of incubation of control microRNA with G2-SN15-PEG 10 µM and G3-SN31-PEG 5 µM dendrimers. b Percentage of microRNA captured *vs.* miRNA control after 2, 24 or 48 h of incubation. **c** Formation of dendriplexes with anti-HIV-1 microRNAs (hsa-miR-29a-3p, hsa-miR-29b-3p, hsa-miR-92a-3p, hsa-miR-133b and hsa-miR-149-5p) and G2-SN15-PEG (10 µM) and G3-SN31-PEG (5 µM) dendrimers after 2 h of incubation. **d** Percentage of microRNA captured *vs.* miRNA control after 2 h of incubation. **e** Formation of dendriplexes with anti-HIV-1 microRNAs and G2-SN15-PEG (5 µM) and G3-SN31-PEG (1 µM) dendrimers. **f** Percentage of microRNA captured *vs.* miRNA control

First of all, whether unlabeled or FITC-labelled dendrimers could form dendriplexes with the studied miRNAs, were explored. Dendrimers were used at the maximal non-toxic concentrations and dendrimers-miRNAs complexes were analyzed using 2% agarose gel. After 2 h of incubation, G2-SN15-PEG and G3-SN31-PEG dendrimers efficiently bound 99% of miRNA with stable binding up to 48 h (Fig. 3a, b). When dendrimer concentrations were lowered to match maximal non-toxic concentrations for U87MG-CD4^+^CCR5^+^ cells, similar results were found. G2-SN15-PEG and G3-SN31-PEG dendrimers bound around 90% and 80% of siRNAs, respectively, confirming that both dendrimers can perform and efficient and stable bond with these miRNAs (Fig. 3c–f).

### Heparin competition and RNase protection assays

**Fig. 4:**
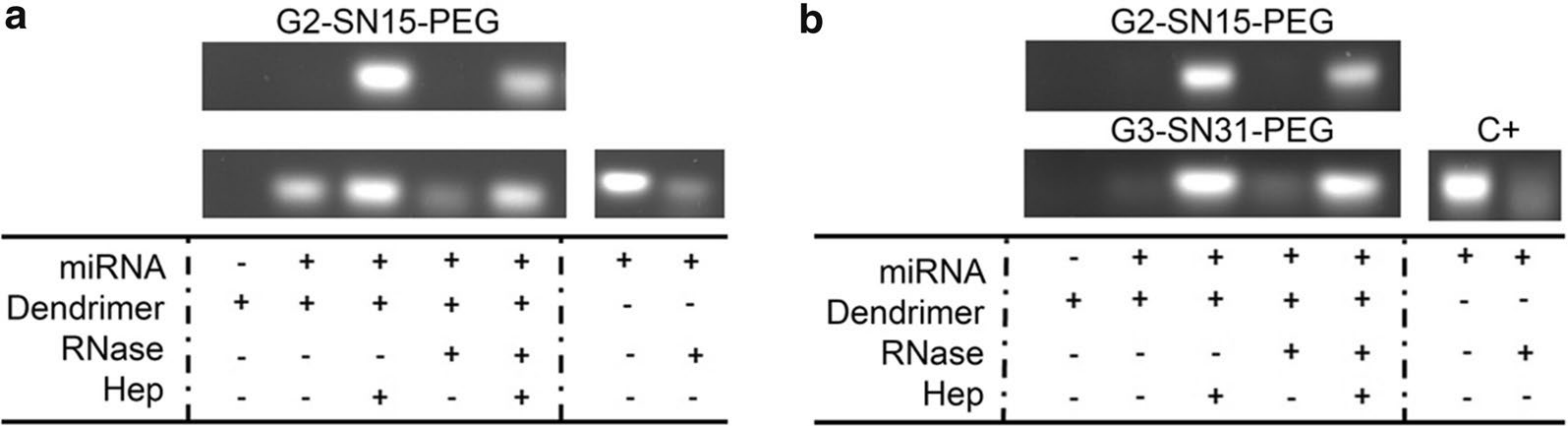
Heparin competition and RNase protection assays. Dendriplexes were prepared with miRNA control 2 h before being treated with heparin and/or RNases. Dendrimers concentrations were **a** G2-SN15-PEG 10 µM and G3-SN31-PEG 5 µM or **b** G2-SN15-PEG 5 µM and G3-SN31-PEG 1 µM

We first analyzed whether miRNAs were released by conducting heparin competition assays. miRNAs were released after heparin treatment, demonstrating that miRNAs could be released from the dendrimer. We also analyzed whether miRNA dendrimer binding protected miRNAs from RNase-mediated degradation, showing that dendrimers confer protection since miRNAs were completely recovered after treatment with RNases followed by heparin (Fig. 4a, b).

Dendriplexes were prepared with miRNA control 2 h before being treated with heparin and/or RNases. Dendrimers concentrations were (a) G2-SN15-PEG 10 µM and G3-SN31-PEG 5 µM, or (b) G2-SN15-PEG 5 µM and G3-SN31-PEG 1 µM.

### Zeta potential and dynamic light scattering

To evaluate how miRNAs affects the properties of the dendrimer we studied the surface charge and stability by measuring the particle zeta potentials (ZP) and the particle size distribution by dynamic light scattering (DLS).

The positive values obtained from the study of the surface charge by ZP measurements confirmed the cationic density of the periphery of the nanoparticles. As shown in Table 2, G2-SN15-PEG dendrimer presents the highest potential value (52,5 mV), which entails high electrical stability. For its part, G3-SN31-PEG presents a moderate value (30,24 mV), which still represents good stability.

**Table 2:** ZP values (mV) for dendrimers and dendriplexes formed with G2-SN15-PEG and G3-SN31-PEG and hsa-miR-29a-3p

Compound	Zeta potential (mV)
G2-SN15-PEG	52.5 ± 0.42
G2-SN15-PEG + hsa-miR-29a-3p	49.45 ± 1.62
G3-SN31-PEG	30.6 ± 0
G3-SN31-PEG + hsa-miR-29a-3p	32.24 ± 2.37

The formation of dendriplexes with hsa-miR-29a-3p led to a change in the ZP values of both dendrimers. More precisely, there is a decrease of the potential value when complexing G2-SN15-PEG dendrimer with the miRNA, whereas there is an increase of the value when forming the dendriplex from G3-SN31-PEG which suggests that the correct formation of the dendriplex took place.

Results obtained from the study of the particle size distribution by DLS (Table 3) suggest aggregation or self-assembly for G2-SN15-PEG (302,95 nm). On the contrary, much lower values were obtained for G3-SN31-PEG dendrimer (4,93 nm) indicating that this one could be described as a single molecule. As for hsa-miR-29a-3p, its size was found with low aggregation degree (14 nm). In terms of dendriplexes formed with G2-SN15-PEG or G3-SN31-PEG dendrimers and hsa-miR-29a-3p, both presented moderate aggregation values (around 30 and 40 nm, respectively). All these values supported with data obtained in electrophoresis assays, confirm the correct formation and stability of complexes.

**Table 3:** Measure of hydrodynamic size for dendrimers and dendriplexes formed with G2-SN15-PEG, G3-SN31-PEG and hsa-miR-29a-3p

Compound	Hydrodynamic size (diameter, nm)
G2-SN15-PEG	302.95 ± 14.78
G2-SN15-PEG + hsa-miR-29a-3p	30.19 ± 2.88
G3-SN31-PEG	4.93 ± 0.26
G3-SN31-PEG + hsa-miR-29a-3p	41.1 ± 2.88
hsa-miR-29a-3p	14.42 ± 4.16

### Internalization assay of the dendrimers

**Fig. 5:**
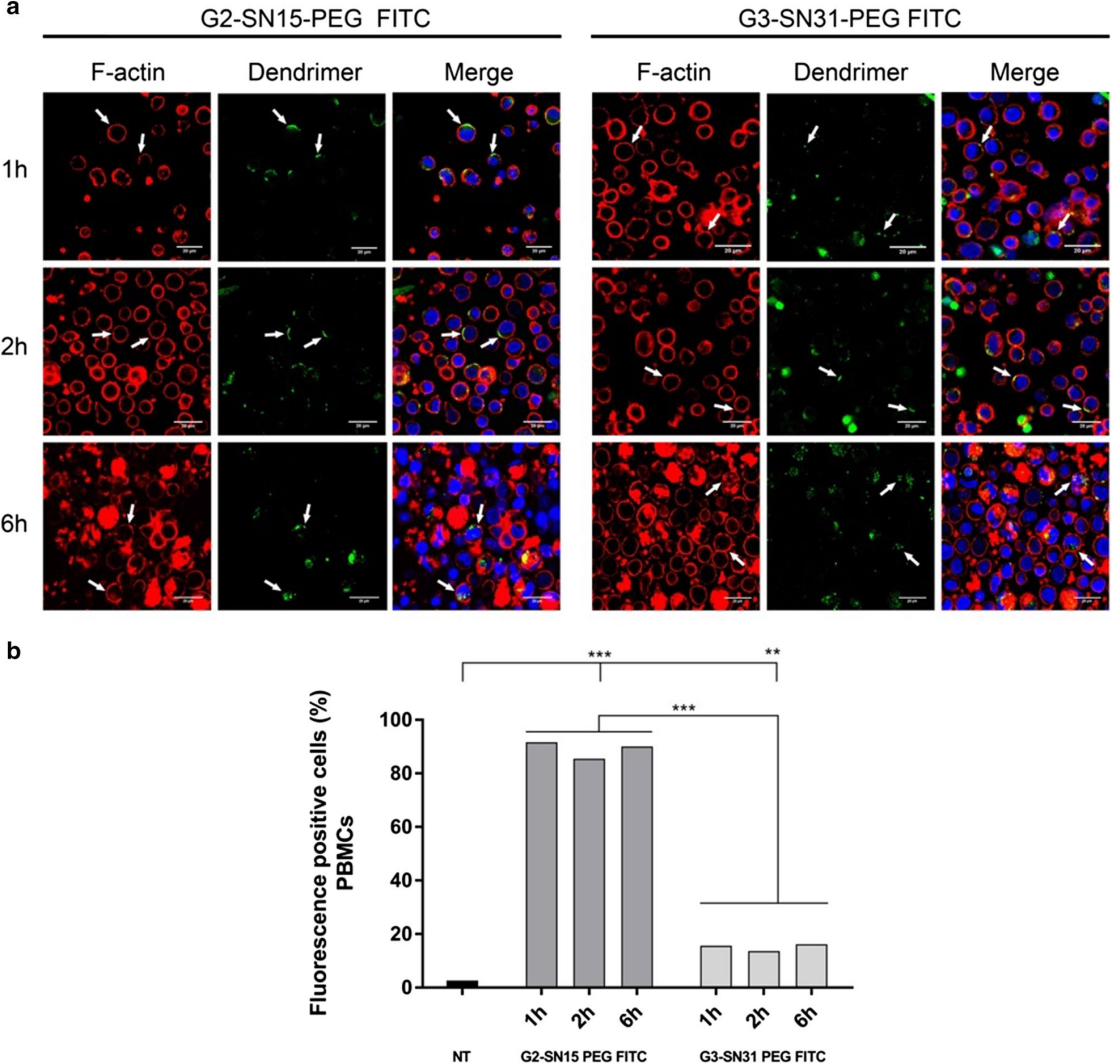
Internalization of cationic carbosilane dendrimers in PBMCs. Confocal microscopy images of PBMCs treated for 1, 2 or 6 h with 5 µM G2-SN15-PEG FITC-dendrimer and 1 µM G3-SN31-PEG FITC-dendrimer (**a**). PBMCs were fixed and stained with Phalloidin (red, actin filaments) and DAPI (blue, nucleus). Confocal images of random field (**b**). Entry of FITC labeled dendrimers into PBMCs observed by flow cytometry at 1, 2 and 6 h post-treatment. *DAPI* 4′,6-Diamidino-2-phenylindole dihydrochloride

The progressive entry of G2-SN15-PEG FITC and G3-SN31-PEG FITC dendrimers in PBMCs and U87MG-CD4^+^CCR5^+^ cell line was determined by confocal microscopy and flow cytometry. For confocal microscopy cells were treated with dendrimers, (5 and 1 µM for PBMCs or 1 and 0.5 µM for U87MG-CD4^+^CCR5^+^ cell line), for 1 h, 2 h or 6 h. Results show a notable difference in the uptake of both dendrimers in CD3 positive cells. In terms of G2-SN15-PEG FITC, there was no differences in the uptake among the studied times, after 1 h of incubation around 90% of PBMCs were positive for dendrimer presence inside the host cell and differences were not detected in the entry percentage at 1, 2 or 6 h. Likewise, when PBMCs were treated with G3-SN31-PET FITC no differences in the uptake at different time points were detected. However, this G3-SN31-PEG FITC showed a percentage of positivity much lower than the previous dendrimer, only around 16% of positive cells (Fig. 5a, b).

**Fig. 6:**
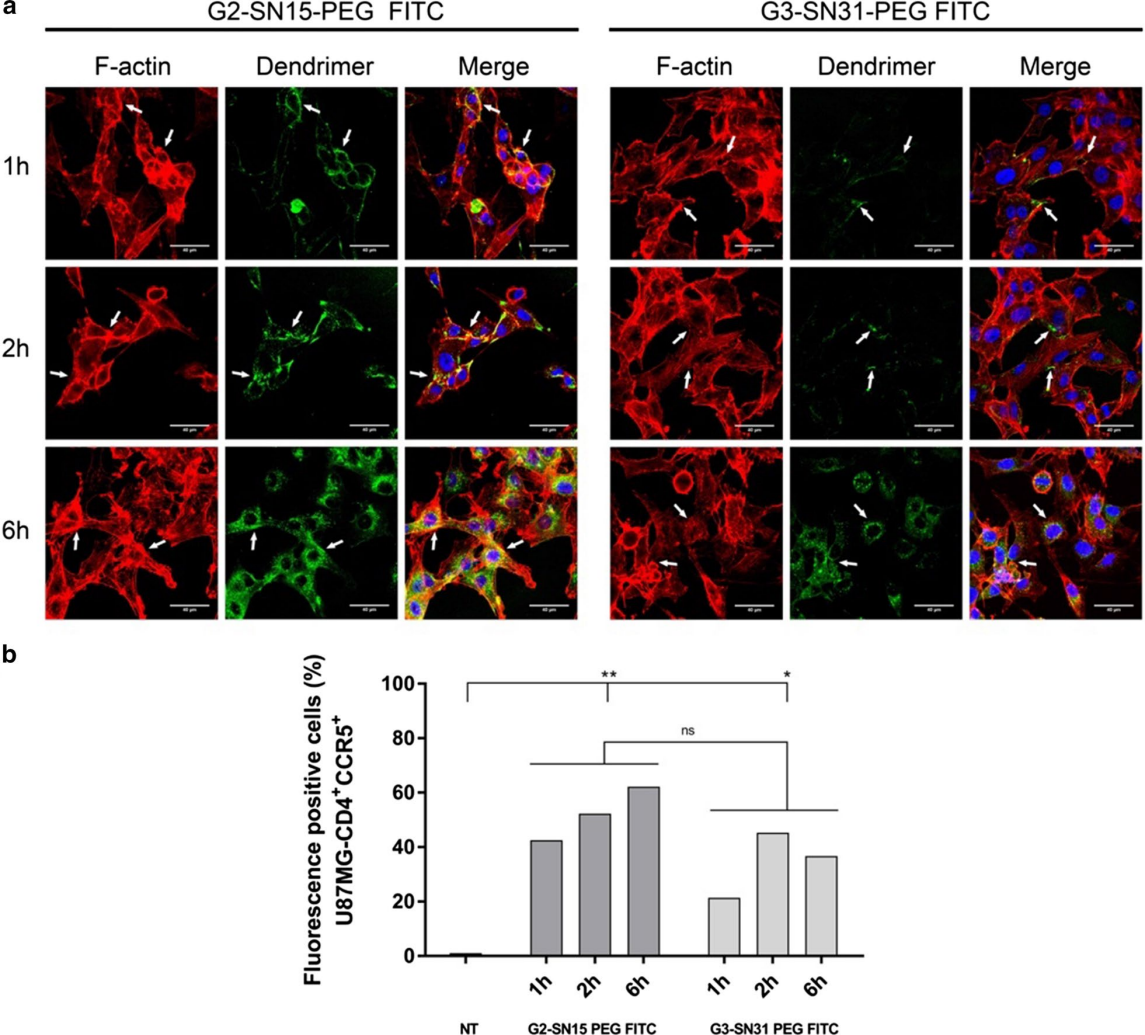
Internalization of cationic dendrimers in U87MG-CD4^+^CCR5^+^ cells. Confocal microscopy images of U87MG-CD4^+^CCR5^+^ cells treated for 1, 2 or 6 h with G2-SN15-PEG FITC 1 µM and G3-SN31-PEG FITC 0.5 µM (green) FITC-labeled dendrimers. Cells were fixed and stained with Phalloidin (red, actin filaments) and DAPI (blue, nucleus). **a** Confocal images of random field. **b** Entry of FITC labeled dendrimers into PBMCs observed by flow cytometry at 1, 2 and 6 h post-treatment. *DAPI* 4′,6-Diamidino-2-phenylindole dihydrochloride

U87MG-CD4^+^CCR5^+^ cells were treated with G2-SN15-PEG FITC 1 µM or G3-SN31-PEG FITC 0.5 µM and the internalization was again studied by confocal microscopy (Fig. 6a) and flow cytometry (Fig. 6b). In this case we can observe again a differential entry between G2-SN15-PEG FITC and G3-SN31-PEG FITC dendrimers and an increase in the number of positive cells along the selected time points. After 1 h of incubation, both dendrimers were found in the outside part of the cell membrane, due to the fact that both dendrimers co-localize with F-actin and flow cytometry indicated that 40% of cells were positive for G2-SN15-PEG FITC whereas only 20% of cells where positive for G3-SN31-PEG FITC. However, after 2 h of incubation we observed a notable increase inside cells and positivity values raised to 50 and 45%, respectively, suggesting that both dendrimers need at least 2 h for internalize into U87MG-CD4^+^CCR5^+^ cells.

Our results indicate that both G2-SN15-PEG FITC and G3-SN31-PEG FITC dendrimers are capable of entering inside of PBMCs and U87MG-CD4^+^CCR5^+^ cells in less than 6 h, showing that both dendrimers could be used as delivery systems.

### HIV-1 inhibition activity of the dendriplexes

**Fig. 7:**
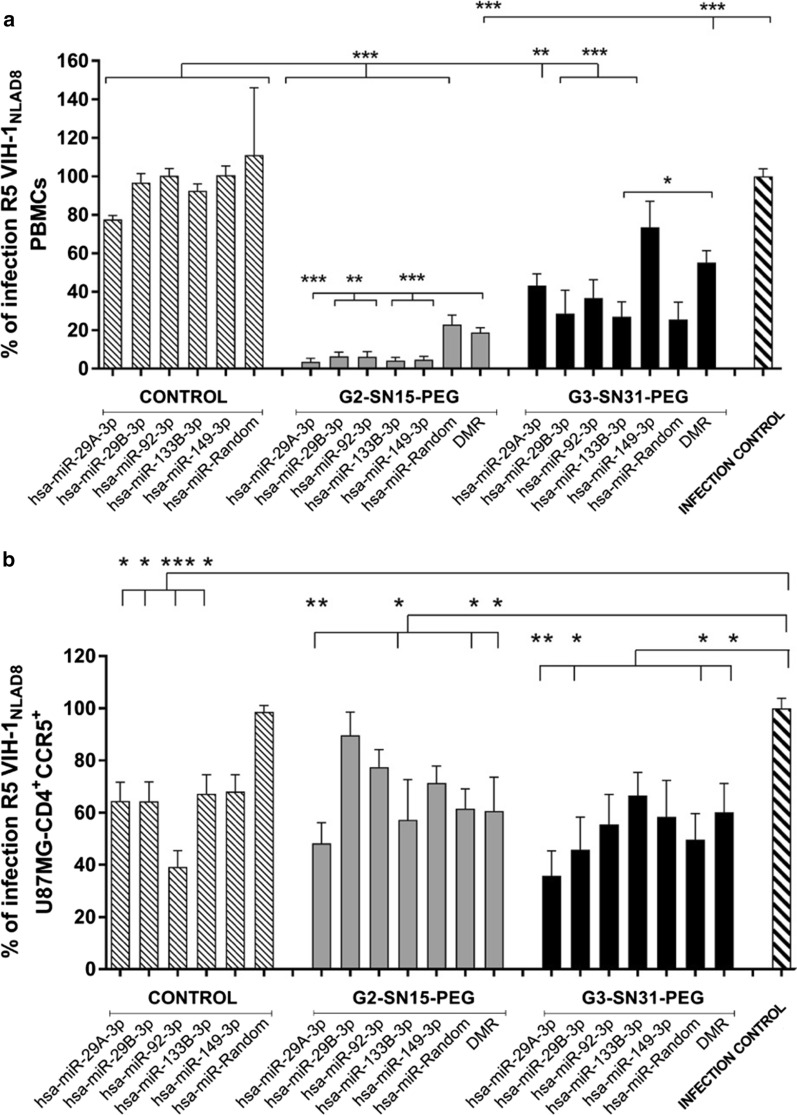
HIV-1 inhibition of dendriplexes. Quantification of R5-HIV-1 infection in **a** PBMCs and **b** U87MG-CD4^+^CCR5^+^ cells treated with dendriplexes. Infection was measured by titration of supernatants on TZM.bl cell line. Infection was inferred from the measurement of luciferase activity 48 h post-titration and was represented as fold change of percentage from HIV-1 infection control. Dendriplexes were formed with non-labeled G2-SN15-PEG or G3-SN31-PEG cationic dendrimer and control or anti-HIV-1 microRNAs (hsa-miR-29a-3p, hsa-miR-29b-3p, hsa-miR-92a-3p, hsa-miR-133b and hsa-miR-149-5p). Data are represented as mean ± SD of three individual experiments performed in triplicate. DMR: dendrimer. (*p < 0.05; **p < 0.01; ***p < 0.001)

To determine the anti-HIV-1 activity of dendriplexes, inhibition experiments were performed using non-labelled dendrimers, with the objective that these dendrimers could be used as a possible therapy. PBMCs or U87MG-CD4^+^CCR5^+^ cells were infected with R5-HIV-1NL_(AD8)_ and treated with dendriplexes after 2 h of incubation of G2-SN15-PEG or G3-SN31-PEG dendrimer and the anti-HIV-1 miRNAs at concentrations of 10 and 5 µM for PBMCs or 5 and 1 µM for U87MG-CD4^+^CCR5^+^, respectively. Then, 72 h after R5-HIV-1NL_(AD8)_ infection, supernatants of PBMCs or U87MG-CD4^+^CCR5^+^ cells were collected and titrated on TZM.bl cells to quantify R5-HIV-1NL_(AD8)_ infection by luciferase activity measurements. Non-treated samples were used as uninfected control. The treatment of PBMCs with G2-SN15-PEG or G3-SN31-PEG dendrimer caused around 80 or 45% of HIV-1 reduction, regarding the control of infection. This inhibition was even greater when PBMCs were treated with dendriplexes formed with anti-HIV-1 miRNAs and both G2-SN15-PEG or G3-SN31-PEG dendrimer showing a significant increase of inhibition reaching values of 96 and 73% reduction of HIV-1 infection, respectively (Fig. 7a).

Treatment of U87MG-CD4^+^CCR5^+^ cells (Fig. 7b) with G2-SN15-PEG FITC 1 µM or G3-SN31-PEG dendrimer caused a significant inhibition of HIV-1 infection, around 40%, in agreement with previously mentioned in the bibliography.34 Moreover, the treatment with some of the dendriplexes produced an increase of HIV-1 inhibition, for example hsa-miR-29A-3p with G2-SN15-PEG or G3-SN31-PEG dendrimer. As expected, this increase was not significant regarding the inhibition resulting from the treatment with only G2-SN15-PEG or G3-SN31-PEG dendrimer, suggesting that dendriplexes cause a significant inhibition of R5-HIV-1 infection only in specific cell lines.

## Discussion

Combinations of antiretroviral therapies have been a major step towards controlling HIV-1 infection, suppressing viral load to undetectable levels, and reducing mortality and morbidity associated with HIV-1 infection [38, 39]. These treatments are unable to overcome the main problems that infection still entails: the existence of stable viral reservoirs and appearance of resistant strains [40–43]. The use of small RNAs is a powerful approach to target these problems, since a broad range of miRNAs has a negative impact on HIV-1 replication [28–30]. The delivery of miRNAs to infected cells constitutes a boundary for its efficient application. Nanotechnology is a strategy to address this issue, since it has previously shown to efficiently deliver small RNA molecules or small miRNAs [12–14]. Our group has carried out wide research about this topic, specifically related to the use of dendrimers in the context of HIV-1 infection [18, 23, 44, 45]. These three-dimensional hyperbranched nanostructures have modifiable edges which promote, for example, the formation of dendriplexes through the interplay among these edges, when positively charged, and the negatively charged backbone of small RNAs. We have analyzed the potential therapeutic effect of the use of four novel cationic dendrimers as delivery systems for different anti-HIV-1 miRNAs to control HIV-1 progression in PBMCs and U87MG-CD4^+^CCR5^+^ cells.

The initial phase of this study was to determine the biocompatibility of dendrimers on PBMCs and U87MG-CD4^+^CCR5^+^ cell line. Cationic dendrimers interact with cell surface, which is negatively charged, producing nanoscale pores in the membrane increasing its permeability. This interaction affects the integrity of membrane depending on different characteristics of dendrimers, such as molecular size or presence of fluorescent markers that could lead to cell death [46]. Therefore, our four dendrimers were conjugated with PEG to mask some cationic groups reducing the toxicity and immunogenicity, and improving solubility, drug loading and drug delivery [32].

Cytotoxicity analysis, performed by MTT assay showed that G3-SN31-PEG FITC dendrimer had the highest toxicity rates followed by G2-SN15-PEG FITC, G3-SN31-PEG and G2-SN15-PEG dendrimers, respectively. As expected, our results show that larger molecular sizes and introduction of fluorescent labels enhance the cytotoxicity of compounds. Studies of genotoxicity, based on SCE assay, showed that non-labelled dendrimers can be used in vivo, did not cause genetic toxicity. It is important to note that these dendrimers could be not used if they affect the host genome and produced genetic changes. The fact that selected miRNAs are naturally present on host cell makes unnecessary to study genotoxicity of these dendrimers.

The capability of different PEGylated cationic dendrimers to complex with various miRNAs to form dendriplexes using the maximum non-toxic working concentrations was studied. Dendriplexes were formed by electrostatic interactions among positively charged functional groups at periphery of dendrimers and negatively charged backbone of small miRNAs [47]. G2-SN15-PEG and G3-SN31-PEG dendrimers can efficiently form dendriplexes after 2 h of incubation with different miRNA in agarose gel electrophoresis.

We also analyzed whether dendrimers conferred protection to miRNAs from RNase-mediated degradation and if dendrimer-miRNA bound could be disengaged in order to enable its binding mRNA. RNase protection assay showed that miRNAs are completely recovered from dendriplex after incubation with RNases, indicating that dendrimer-miRNA complex protects miRNAs from degradation. Moreover, dendriplexes can be completely dissociated when dealing with a binding site competitor, such as heparin. Moreover, dendrimer-miRNAs complex will act in case of piercing of plasmatic membrane of host cell, because heparin assay recreates this situation caused by the disparity in the number of charges due to different environments between cytoplasm and extracellular fluids, thus proving that dendrimers release complexed miRNA when internalizing in the host cell.

To evaluate how the addition of the miRNAs affects the properties of the dendrimer we studied the surface charge and stability by measuring the particle zeta potentials (ZP) and the particle size distribution by dynamic light scattering (DLS). Results indicated that both dendrimers are stable, and their surface charges are positive in solution. In addition, results showed a fall of the ZP value in third-generation dendrimer (G3-SN31-PEG) compared to second-generation dendrimer (G2-SN15-PEG), which can be explained on the basis of a more effective wrapping of the PEG moiety, because PEGylation reduces charge density, as previously described elsewhere [48]. When studying the effect of the complexation of miRNAs, values showed a small change of ZP values, confirming the formation of the dendriplexes without affecting the stability of the particles.

In terms of the particle size distribution, G2-SN15-PEG dendrimer was found to form aggregates probably due to a reorganization over time owing to interactions between different molecules when it is in solution. On the other hand, G3-SN31-PEG dendrimer was described as a single molecule and hsa-miR-29a-3p also presented low aggregation values. It would be expected for the hydrodynamic size of the complexes to be superior to that of the individual dendrimers as discrete particles. However, when forming the dendriplex with G2-SN15-PEG dendrimer aggregation values were lower, which could be explained because the introduction of the miRNA into the medium leads to more favorable electrostatic binding and contributes to more stable systems in solution without the need for aggregation by the dendrimer. The nanoconjugate formed with G3-SN31-PEG dendrimer and hsa-miR-29a-3p presented the expected increase in the hydrodynamic size after the formation of the complex. Changes in particle size distribution of the dendrimers, along with data obtained in electrophoresis assays, confirm the correct formation and stability of complexes.

The internalization of both FITC-labelled dendrimers in PBMCs and U87MG-CD4^+^CCR5^+^ cells confirm that these dendrimers could be used as carriers. Cationic dendrimers cross cell membranes with different effectiveness depending on dendrimer properties such as size or surface charge and cellular features such as and membrane chemical composition. These elements determine the affinity with which dendrimers are bound to cell surface: higher amounts of positive charges entail stronger interactions that lead to longer resident times on membrane and, thus, slower internalization [49]. This fact has been reflected in our results, where we can observe a higher internalization of G2-SN15-PEG FITC dendrimer in both cell lines studied compared to G3-SN15-PEG FITC dendrimer, probably due to a higher affinity with cell surface produced by the presence of more positive charges being a bigger molecule.

Regardless of this result, both dendrimers are able to internalize into host cells in less than 6 h and these two dendrimers present a resembling behavior inside the cells, showing that both dendrimers can be used as delivery systems.

After demonstrating that G3-SN31-PEG FITC and G2-SN15-PEG FITC dendrimers form stable dendriplexes that confer protection to different miRNAs and that can be efficiently delivered, we studied their anti-HIV-1 activities. This activity was tested in R5-HIV-1NL_(AD8)_ infected PBMCs and U87MG-CD4^+^CCR5^+^ cells to determine whether the conjugation of both G3-SN31-PEG and G2-SN15-PEG non-labelled dendrimers with cellular anti-HIV-1 miRNAs improved the therapeutic effect. Our results showed that both dendrimers are able to significantly inhibit HIV-1 infection in both PBMCs and U87MG-CD4 + CCR5 cells. However, inhibition with dendriplexes was only significantly improved when treating PBMCs suggesting a specific behavior depending on cell lineage. The selected miRNAs cannot decrease the HIV-1 infection by themselves in PBMCs. They need the formation of complexes with dendrimers. The main objective was to prove that these novel cationic dendrimers can be used as delivery agents in order to replace current methods and develop a safer method with possible in vivo perspectives. Our results could fill the gap between the in vitro delivery researches and the possibility of implementing this technology in clinic, developing a new “from bench to patient” safe and effective technology.

## Conclusion

In conclusion, the PEGylated carbosilane dendrimers G2-SN15-PEG and G3-SN31-PEG are valid delivery systems for miRNAs. Formation of dendriplexes provides miRNAs with protection against degradation and enables delivery to different cells. These dendriplexes specifically and significantly improve the anti-R5-HIV-1 activity of miRNAs, being good candidates to be used therapeutically to address the main problems that the HIV-1 infection still entails.

## Methods

### Cell lines

TZM.bl cell line (NIH AIDS Research and Reference Reagent Program, Germantown, MD, USA) is a human cervical epithelial carcinoma cell (HeLa cell line), that expresses CD4 receptor and CCR5 co-receptor and contains β-galactosidase and luciferase genes under control of long terminal repeat (LTR) regions of HIV-1 promoter [50]. U87MG-CD4^+^CCR5^+^ (NIH AIDS Research and Reference Reagent Program, Germantown, MD, USA) is a human astrocytoma (glioblastoma) cell line derived from U87MG to express CD4 receptor and CCR5 and CXCR4 co-receptors.

TZM-bl cell line was cultured in Dulbecco’s Modified Eagle’s Medium (DMEM) (Biochrom GmbH, Berlin, Germany) supplemented with 5% heat-inactivated fetal bovine serum (FBS) (Biochrom GmbH, Berlin, Germany), 2 mM L-glutamine (Lonza, Base, Switzerland) and a cocktail of antibiotics (125 mg/ml ampicillin, 125 mg/ml cloxacillin and 40 mg/ml gentamicin (Normon, Madrid, Spain)) at 37ºC with 5% CO2. U87MG-CD4^+^CCR5^+^ cell line was maintained in DMEM supplemented with 10% FBS, 2 mM L-glutamine and the aforementioned antibiotic cocktail at 37ºC with 5% CO2.

### Primary cells

Buffy coats, acquired from healthy anonymous donors from the Transfusion Centre of Madrid (Madrid, Spain) following the current legislation, were used to obtain PBMCs on a Ficoll-Hypaque density gradient (Rafer) according to standard procedures of Spanish HIV HGM BioBank [51].

After the isolation, PBMCs were cultured (5 × 10^6^/mL) in RPMI 1640 (Biochrom GmbH, Berlin, Germany) supplemented with 10% FBS, 2 mM L-glutamine, the aforesaid cocktail of antibiotics, 60 IU/mL of interleukin-2 (IL-2, Bachem, Budendorf, Switzerland) and 2 µg/mL of phytohemagglutinin (PHA) (Remel, Dartford, Kent, UK).

### Viral isolates

Viral stock of CCR5-tropic R5-HIV-1NL_(AD8)_ laboratory isolate was obtained by transient transfection with pNL_(AD8)_ plasmid (NIH AIDS Research and Reference Reagent Program, Germantown, MD, USA) into HEK- 293 T cell line (ATCC, Manassas, VA, USA). Viral stock was clarified by centrifugation and the viral titter was later evaluated using an HIV-1 p24gag enzyme-linked Immunosorbent assay (ELISA) kit (INNOTEST HIV, Antigen mAb, Innogenetics, Ghent, Belgium), as previously reported [52].

### MicroRNAs

Five cellular miRNAs reported to have anti-HIV-1 activity were selected based on their homology with HIV-1 genome: hsa-miR-29a-3p, hsa-miR-29b-3p, hsa-miR-92a-3p, hsa-miR-133b and hsa-miR-149-5p (QUIAGEN, Hilden, Germany) [33, 34]. We also selected one miRNA with random sequence but similar length and no activity against HIV-1 as a negative control (QUIAGEN, Hilden, Germany). Sequence, HIV-1 genome target and effect on HIV-1 replication are shown in Table 4. Stock solutions of miRNAs (66,7 µM) and working concentrations (6670 nM) were prepared in nuclease-free water (Promega, Madrid Spain) and stored at − 20ºC.

**Table 4:** Sequence, HIV-1 target and effect on HIV-1 replication of selected anti-HIV-1 microRNAs and control microRNA

Name	Sequence	HIV-1 genome target	Effect on HIV-1 replication
miR-29a-3p	UAGCACCAUCUGAAAUCGGUUA	*Nef*/3’UTR	Negative
miR-29b-3p	UAGCACCAUUUGAAAUCAGUGUU	*Nef*/3’UTR	Negative
miR-92a-3p	UAUUGCACUUGUCCCGGCCUGU	*Pol*	Negative
miR-133b	UUUGGUCCCCUUCAACCAGCUA	*Env*	Negative
miR-149-5p	UCUGGCUCCGUGUCUUCACUCCC	*Gag*	Negative
miR-Control	UCACCGGGUGUAAAUCAGCUUG	-	None

### Dendrimers and reagents

**Fig. 8:**
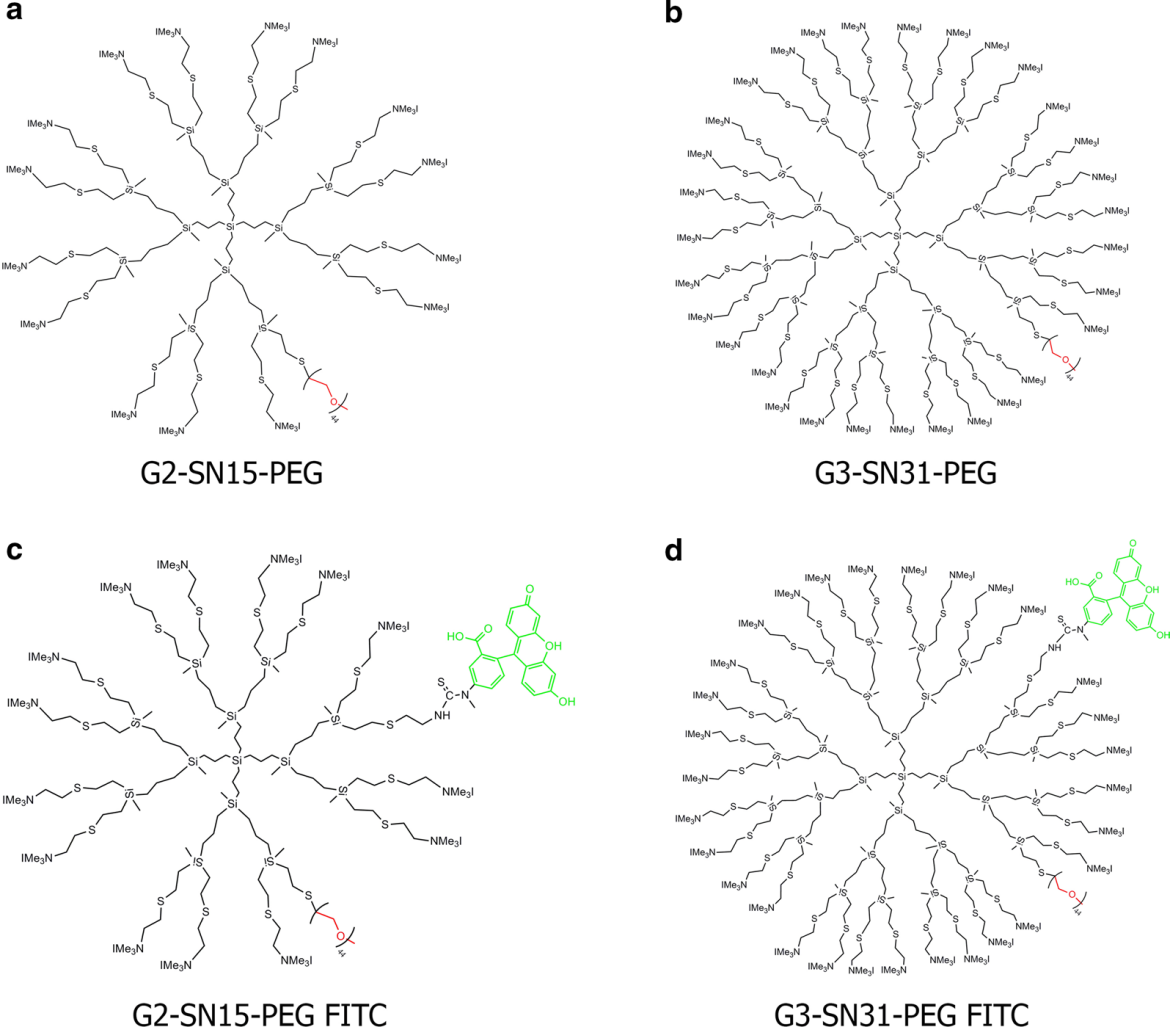
Schematic representation of PEGylated cationic dendrimers. Molecular representation of **a** G2-SN15-PEG, **c** G2-SN15-PEG FITC (second generation dendrimers), **b** G3-SN15-PEG and **d** G3-SN31-PEG FITC (third generation dendrimers)

PEGylated cationic dendrimers G2-SN15-PEG and G3-SN31-PEG and their FITC-labelled forms were synthesized and tested according to the methods described by Dendrimers for Biomedical Applications Group of University of Alcalá (Madrid, Spain). Stock solutions of dendrimers (1 mM) and subsequent dilutions for working concentrations were prepared in nuclease-free water (Promega, Madrid Spain). The schematic structures of PEGylated cationic carbosilane dendrimers are represented in Fig. 8.

### Mitochondrial activity assay

Mitochondrial activity was determined by the 3-(4–5-dimethylthiazol-2-yl)-2,5-diphenyltetrazolium bromide (MTT) assay (Sigma, St Louis, MO, USA) following manufacturer’s instructions to establish the mitochondrial toxicity of the dendrimers. Briefly, 2 × 10^5^ PBMCs or 7.5 × 10^3^ U87MG-CD4^+^CCR5^+^ cells/well were seeded in 96-well plates and treated with different concentrations of dendrimers for 72 h. After incubation, culture medium was discarded and a 200 μL of a mixture of MTT/Opti-MEMTM solution (1:11) (Thermo Fisher Scientific, Waltham, MA, USA) was added. After 2 h, the reaction was stopped by removing the solution and dissolving the formazan crystals in 200 μL of dimethyl sulfoxide (DMSO, Honeywell, Charlotte, NC, USA) and absorbance was recorded in a Synergy 4 plate reader (BioTek, Winooski, VT, USA) at 490 nm. Ten per cent DMSO was used as cellular death control and culture medium as non-treated control. All measurements were performed in triplicate three times.

### Genotoxicity of dendrimers

To assess the genotoxicity of non-labelled G2-SN15-PEG and G3-SN31-PEG dendrimers sister chromatids exchange (SCE) assay was performed in PBMCs. Cells were treated with G2-SN15-PEG 10 µM or G3-SN31-PEG 5 µM for 72 h. Radiation at 1 Gy was used as genotoxic positive control and non-treated cells were used as control of genetic viability. Cell cultures were then treated with 25 µM bromodeoxyuridine (BrdU) (Thermo Fisher Scientific, Waltham, MA, USA) to monitor second divisions. After 70 h, 0.1 µM/mL colcemid (Thermo Fisher Scientific, Waltham, MA, USA), a mitosis inhibitor, was added to cultures and, 2 h after, metaphases were extended and analyzed by fluorescence plus Giemsa (FPG). Briefly, cells were centrifuged 10 min at 600 g and the supernatant was discarded and 10 mL of 5.6 µg/mL potassium chloride (Merck KGaA, Darmstadt, Germany) was added. Cells were then centrifuged 10 min at 600 g, the supernatant was discarded, and the pellet was fixed with methanol/glacial acetic acid (3:1) (PanReac AppliChem, Madrid, Spain). This procedure was repeated 3 more times and the resulting metaphases were dropped over slides (Thermo Fisher Scientific, Waltham, MA, USA).

After 48 h maturing at 56ºC, slides were immersed in Hoechst solution (Sigma, St Louis, MO, USA) for 30 min, washed with water and immersed in McIlvaine buffer (0.55 mL citric acid plus 19.45 mL di-Sodium hydrogen phosphate) (Merck KGaA, Darmstadt, Germany), for 40 min in a thermostatic plate at 56ºC 5 cm away from UV light. Slides were then washed and stained with Giemsa (Merck KGaA, Darmstadt, Germany) for 7 min. Images were obtained with a Leica DM 5000 B Microscope and analyzed with Cytovision® software (Leica, Wetzlar, Germany).

### Dendriplexes formation

Dendrimer-miRNA complexes were formed mixing the maximum non-toxic concentration of the different cationic dendrimers with 100 nM of the desired miRNAs in nuclease-free water (Promega, Madrid, Spain). The formation and stability of dendriplexes were evaluated by agarose gel electrophoresis after 2, 24 and 48 h of incubation at room temperature (RT). Samples were treated with Blue/Orange Loading Dye 6X (Promega, Madrid, Spain) and loaded on a gel with 2% agarose (Sigma, St Louis, MO, USA) and 0.01% GelRed® (Biotium, Fremont, CA, USA) in Tris–acetate-EDTA (TAE) buffer (PanReac AppliChem, Darmstadt, Germany) at 120 mV for 40 min on a PowerPac Universal power supply (Bio-Rad Laboratories, Hercules, CA, USA).

### Heparin competition assay and RNase protection

Heparin exclusion and RNase protection assays were performed to test the capacity to disengage miRNAs from the complex and whether the formation of dendriplexes confers protection against RNases, respectively. Dendriplexes were formed within 2 h of incubation and were treated with 0.2 UI/µL of heparin (Lab Ramón Sala, Barcelona, Spain) for 5 min at RT and/or 1 µg/µL of RNase (Promega, Madrid, Spain) for 10 min at RT and loaded as described before.

### Zeta potential and dynamic light scattering

Surface charge and stability of the dendrimers and dendriplexes were studied by measuring the zeta potential (ZP) and their distribution analyzed measuring the hydrodynamic size (diameter) by dynamic light scattering (DLS).

Dendrimer and dendriplexes samples were prepared in nuclease-free water (Promega, Madrid, Spain) using the maximum non-toxic concentrations (G2-SN15-PEG 10 µM and G3-SN15-PEG 5 µM) and hsa-miR-29A-3p (100 nM). For ZP, samples were loaded into folded capillary cells and measured using a voltage of 100 V. For DLS, samples were loaded into quartz cuvettes with pathlength 10 mm. Measurements were performed at 25ºC with a Zetasizer Nano ZS spectrometer (Malvern Instrument, Malvern, UK). Three measurements with fourteen cycles were made for each sample.

### Confocal microscopy

Internalization of the dendrimers into cells was analyzed by confocal microscopy with a Leica TSC SPE Confocal Microscope (Leica, Wetzalar, Germany). PBMCs were seeded at a density of 1 × 10^6^ cells/well in 24-well plates and U87MG-CD4^+^CCR5^+^ cells were seeded at 7.5 × 10^3^ cells in 12 mm circle cover slips (Thermo Fisher Scientific, Waltham, MA, USA) pre-treated for 24 h with poly-L-Lysine (Sigma, St Louis, MO, USA). Cells were treated with the fluorescent dendrimers for 1 h, 2 h or 6 h at 37ºC. After incubation, handling PBMCs as suspension cells and U87MG-CD4^+^CCR5^+^ as adherent cells, cells were rinsed twice with 3% bovine serum albumin (BSA, Sigma, St Louis, MO, USA) phosphate buffered saline (PBS, Lonza, Base, Switzerland), fixed with 4% paraformaldehyde (PFA, Panreac, Barcelona, Spain) and permeabilized with 0.1% Triton 100X (Sigma, St Louis, MO, USA) for 15 min. Cells were then incubated with Alexa Fluor® 555 Phalloidin (Thermo Fisher Scientific, Waltham, MA, USA) for 1 h at RT for actin labelling and then rinsed with PBS 3% BSA. Lastly, cells were incubated with 4′,6-Diamidino-2-phenylindole dihydrochloride (DAPI, Sigma, St Louis, MO, USA) for nuclear visualization and mounted in microscope slides (Dako, Carpinteria, CA, USA) with fluorescent mounting media (Dako, Carpinteria, CA, USA).

### Flow cytometry

The internalization of FITC labelled dendrimers was confirmed by flow cytometry. Briefly, 1 × 10^6^ PBMCs or 1.5 × 10^4^ U87MG-CD4^+^CCR5^+^ cells/well were seeded in 24-well plates and treated with the fluorescent dendrimers for 1, 2 or 6 h at 37ºC. After incubation, adherent cells were removed by trypsinization and rinsed with PBS 3% BSA. PBMCs were incubated with anti-CD3-PC5 (Beckman Coulter, Brea, CA, USA) for 30 min at RT and then rinsed with PBS 3% BSA. Viability was assessed in both cell lines with 7-Aminoactinomycin D (7-AAD) (Sigma, St Louis, MO, USA) following manufacturer’s instructions in both cell lines. Lastly, cells were fixed with 4% PFA. Measurements were analyzed using Kaluza software (Beckman Coulter, Brea, CA, USA).

### HIV-1 inhibition activity of the dendriplexes

Inhibition experiments were carried out to test the anti-R5-HIV-1 activity of the different dendriplexes. PBMCs were seeded at a density of 1 × 10^6^ cells/well in 24-well plates and U87MG-CD4^+^CCR5^+^ at 7.5 × 10^3^ cells/well in 96-well plates. Afterwards, cells were infected with R5-HIV-1NL_(AD8)_ strain (15 ng p24/10^6^ cells) and treated with the desired dendriplexes previously incubated for 2 h to allow the complex formation. 72 h after the infection, supernatants were collected and titrated or frozen at − 80 ºC.

### Supernatant titration

Viral infectivity was inferred by luciferase activity measurements performed following supernatant titration on TZM.bl cells with the previously collected supernatants. TZM.bl cells were seeded at a density of 1 × 10^4^ cells/well in 96-well plates. Subsequently, medium was removed and replaced with 100 µL of new medium and 100 µL of supernatants from PBMCs or U87MG-CD4^+^CCR5^+^ infected and treated cells. 48 h post-titration, medium was removed, and cells were lysed with 50 µL of the Cell Culture Lysis 5X Reagent (Promega, Madrid Spain) for 30 min at 4ºC. Finally, 25 µL of the lysates were transferred to a white clear bottom plate and 25 µL of Luciferase Assay Reagent (Promega, Madrid, Spain) were added right before reading the luciferase activity in a Synergy 4 plate reader at a 135/200 nm.

### Statistics

GraphPad software Prism v.5.0 (GraphPad Software, San Diego, CA, USA) was used for the different statistical analysis performed. Data with three replicates are displayed as bars ± SD. A p-value of ≤ 0.05 was considered to be statistically significant (*p < 0.05; **p < 0.01; ***p < 0.001).

## Data Availability

Not applicable.
